# Effectiveness of Caprini risk stratification-guided nursing in preventing postoperative deep vein thrombosis among patients with lower limb fractures

**DOI:** 10.1097/MD.0000000000044394

**Published:** 2025-10-03

**Authors:** Shuwei Huang, Yimiao Wang, Lilin Cao, Naikun Gao, Liqun Jia, Ruyue Wang, Chunyan Liu

**Affiliations:** a Department of Critical Care Medicine, The First Affiliated Hospital of Hebei North University, Zhangjiakou, Hebei Province, China.

**Keywords:** Caprini risk assessment, DVT, lower limb fractures, stratified nursing

## Abstract

This retrospective study investigates the clinical impact of risk-stratified nursing care, guided by the Caprini assessment tool, on postoperative deep vein thrombosis (DVT) prevention in patients undergoing lower limb fracture surgery. The study evaluates differences in DVT incidence, time to thrombosis onset, and postoperative functional outcomes between intervention and control groups. We analyzed medical records of patients admitted for lower limb fracture treatment between October 2023 and January 2024. Participants were categorized into 2 cohorts: an intervention group (n = 51) receiving Caprini-based stratified nursing and a control group (n = 69) managed with conventional care. Propensity score matching ensured baseline comparability. Outcome measures included DVT occurrence rates, thrombosis-free survival duration, and functional recovery indicators. The intervention group demonstrated superior outcomes across multiple parameters. DVT incidence was markedly lower in the stratified nursing group (5.9%) compared to controls (17.6%, *P* = .04). Time-to-event analysis revealed significantly prolonged thrombosis-free survival in the intervention group (median 15 vs 10 days, *P* = .03). Subgroup analysis showed particularly pronounced risk reduction in high-Caprini-score patients (10.0% vs 30.0%, *P* = .02). Furthermore, the intervention group achieved significantly better functional recovery scores, including Harris Hip Score, AOFAS Ankle Score, Knee Society Score, and SF-36 Physical Component Summary (*P* < .05). Implementation of Caprini risk stratification-guided nursing protocols effectively reduces postoperative DVT risk, delays thrombosis onset, and enhances functional recovery in lower limb fracture patients. These findings support the clinical adoption of personalized, risk-adapted nursing strategies for postoperative thromboprophylaxis and rehabilitation.

## 1. Introduction

Venous thromboembolism (VTE), primarily including deep vein thrombosis (DVT) and pulmonary embolism (PE), is one of the most serious perioperative complications in patients with lower limb fractures. The mechanism of VTE, as summarized by Virchow, includes 3 key factors: venous stasis, endothelial injury, and a hypercoagulable state.^[1–3]^ DVT specifically refers to the abnormal coagulation of blood within the deep venous lumen, which adheres to the vessel wall and physically narrows the venous lumen, leading to impaired venous return. It predominantly occurs in the lower limbs of adults. Lower limb fractures can cause severe localized pain, which, as 1 of the 5 vital signs, may induce vascular spasm and the release of inflammatory factors. Vascular spasm mechanically alters the diameter of blood vessels and affects blood flow velocity, thereby accelerating thrombosis formation.^[1,4,5]^

Additionally, inflammatory factors play a critical role in the onset and progression of DVT, resulting in a vicious cycle of pain-thrombosis-pain. Lower limb fractures can also cause localized vascular injury and bleeding, leading to hemodynamic changes and placing patients in a hypercoagulable state, further increasing the risk of venous thrombosis. Given that patients with lower limb fractures typically exhibit all 3 of these factors, they are at a significantly higher risk of developing DVT and PE.^[6–8]^

Despite the implementation of effective preventive measures, the incidence of DVT in patients with lower limb fractures remains as high as 2.4% to 6.49%. While there is a general consensus on perioperative DVT prevention in these patients, DVT during the perioperative period continues to be a significant challenge for orthopedic clinicians.^[9–11]^ Therefore, effectively predicting high-risk patients, particularly those at high risk of DVT among lower limb fracture patients, is of paramount importance.

The Caprini risk assessment model, validated and refined through extensive clinical trials, is a simple, practical, cost-effective, and reliable tool that is well-suited for clinical application.^[12]^ We hypothesize that the Caprini risk stratification can more accurately identify high-risk DVT patients following lower limb fracture surgery, thereby enhancing its clinical predictive value. Based on this scoring model, this study aims to evaluate the risk of thrombosis following lower limb fracture surgery and assess its effectiveness and clinical value in identifying high-risk DVT patients.

Additionally, this study will analyze preoperative and postoperative (day 3) plasma fibrinogen and D-dimer levels to explore their value in the early prediction of postoperative DVT complications in patients with lower limb fractures.

## 2. Materials and methods

### 2.1. Study design

This study was approved by the Ethics Committee of the First Affiliated Hospital of Hebei North University. This retrospective study collected clinical data of patients with lower limb fractures treated at our hospital from October 2023 to January 2024. To evaluate the effectiveness of stratified nursing based on the Caprini risk assessment in preventing postoperative DVT, a comparative analysis was conducted between an observation group and a control group. Patients were categorized into the observation group or the control group based on whether they received stratified nursing guided by the Caprini risk assessment. The observation group consisted of 51 patients who had previously received stratified nursing based on the Caprini risk assessment, while the control group included 69 patients with Caprini risk assessment information but who had received standard care.

To ensure baseline comparability between the observation and control groups, propensity score matching (PSM) was utilized. Fifty-one patients from the control group with baseline characteristics matched to those of the observation group were selected, achieving balance in key variables and reducing the influence of confounding bias. Additionally, for subgroup analysis, the observation group was divided into 3 risk categories based on the Caprini risk scores: low-risk group: patients with Caprini scores of 0 to 2, considered to have a low risk of postoperative DVT. Moderate-risk group: patients with Caprini scores of 3 to 4, indicating a moderate risk of postoperative DVT. High-risk group: patients with Caprini scores of 5 or higher, considered to have a high risk of postoperative DVT.

*Inclusion criteria*: This study included patients with lower limb fractures who met the following criteria from October 1, 2023, to January 31, 2024: age ≥ 18 years. Diagnosed with lower limb fractures (including femur, tibia, ankle, etc) confirmed by imaging. Scheduled for surgical treatment (e.g., internal fixation, external fixation, joint replacement). Complete postoperative follow-up records available (including DVT monitoring, complications, hospitalization details, etc). Patients or their family members consented to participate in the study and signed an informed consent form.

*Exclusion criteria*: Patients were excluded if they met any of the following criteria: a history of deep vein thrombosis or pulmonary embolism. Severe internal medical conditions (e.g., liver or kidney failure, malignant tumors, heart failure) or other significant diseases affecting thrombosis or treatment. Pregnant or breastfeeding women. Patients who did not undergo Caprini risk assessment.

### 2.2. Nursing interventions

#### 2.2.1. Stratified nursing based on Caprini risk assessment

Patients were categorized into low-risk, moderate-risk, and high-risk groups according to their Caprini risk assessment scores, and corresponding nursing interventions were implemented based on the risk levels. The specific nursing measures are as follows.

##### 2.2.1.1. Low-risk group (Caprini score 0–2)

Patients in this group are considered to have a low risk of postoperative DVT, and the nursing measures are relatively simplified, focusing primarily on meeting basic nursing needs.

*Early mobilization*: Within 24 hours postoperatively, patients were encouraged to perform in-bed exercises, such as leg lifting and ankle movements. By 2 to 3 days postoperatively, patients could stand at the bedside and gradually increase activity levels, avoiding prolonged bed rest.

*Anticoagulation therapy*: Routine anticoagulation therapy was not administered to low-risk patients unless other clinical indications were present.

*DVT monitoring*: Daily palpation of the lower limbs was conducted to check for signs of swelling, tenderness, or redness indicative of DVT. Ultrasound examinations were performed within 2 to 3 days postoperatively to facilitate early detection of DVT.

*Patient education*: Patients and their families were educated on the risks, preventive measures, and warning signs of deep vein thrombosis (e.g., lower limb swelling, pain, and redness). They were encouraged to remain active and actively participate in postoperative rehabilitation.

##### 2.2.1.2. Moderate-risk group (Caprini score 3–4)

Patients in this group have a moderate risk of postoperative DVT, requiring more proactive nursing interventions, combining pharmacological and non-pharmacological measures for prevention.

*Early mobilization*: Patients were encouraged to perform in-bed exercises within 24 hours postoperatively, gradually increasing activity levels. By 2 to 3 days postoperatively, patients were advised to stand and start walking as early as possible, with an emphasis on weight-bearing activities for the lower limbs.

###### 2.2.1.2.1. Anticoagulation therapy

*Drug selection*: Routine administration of low-molecular-weight heparin (LMWH) or low-dose heparin (e.g., enoxaparin, dalteparin) with dosage adjustments based on the patient’s weight and type of surgery.

*Administration*: Anticoagulants were given via subcutaneous injection once daily for 7 to 10 days, until the patient resumed normal activity and the risk of thrombosis decreased.

*Monitoring*: Regular monitoring of coagulation parameters, such as international normalized ratio and activated partial thromboplastin time.

###### 2.2.1.2.2. Mechanical prevention

Intermittent pneumatic compression (IPC): IPC devices were applied immediately after surgery to enhance lower limb blood circulation. Each session lasted at least 30 minutes, 3 to 4 times daily, until the patient could ambulate.

*Compression stockings*: Compression stockings with 20 to 30 mm Hg pressure were used to facilitate venous blood return and reduce venous stasis.

*DVT monitoring*: Daily palpation of the lower limbs was conducted to check for signs such as swelling, redness, or tenderness. Ultrasound examinations were scheduled within 3 to 5 days postoperatively for early detection of DVT.

*Patient education*: Patients were informed about DVT warning signs (e.g., lower limb swelling and pain) and educated to avoid prolonged sitting or standing. Emphasis was placed on promoting blood circulation in the lower limbs.

##### 2.2.1.3. High-risk group (Caprini score ≥ 5)

For high-risk patients, nursing interventions must be particularly proactive, employing multiple measures to minimize the risk of DVT as much as possible.

###### 2.2.1.3.1. Early mobilization

*Within 24 hours postoperatively*: Initiate in-bed activities, such as ankle rotations and dorsiflexion exercises.

*Within 2 to 3 days postoperatively*: Encourage early bedside standing and walking. Gradually increase activity levels and incorporate weight-bearing exercises for the lower limbs.

*Functional rehabilitation training*: Based on the patient’s recovery, start rehabilitation therapy within 3 to 5 days postoperatively, including physical therapy and joint mobility exercises, to promote blood circulation and functional recovery.

###### 2.2.1.3.2. Anticoagulation therapy

*Drug selection*: Administer LMWH or unfractionated heparin for continuous anticoagulation. Depending on the patient’s condition, choose between subcutaneous injection or intravenous infusion.

*Duration of anticoagulation therapy*: Typically administered for at least 7 to 10 days, continuing until the patient resumes activity and shows no signs of DVT.

*Medication monitoring*: Monitor coagulation function daily to ensure effective anticoagulation. Regularly check parameters such as activated partial thromboplastin time or international normalized ratio to avoid excessive anticoagulation.

*Post-discharge anticoagulation plan*: Continue oral anticoagulants (e.g., warfarin, apixaban) after discharge until the DVT risk is reduced.

###### 2.2.1.3.3. Mechanical prevention

*IPC*: Apply IPC devices postoperatively, 3 to 4 times daily, for 30 minutes per session, until the patient can ambulate and resume normal activities.

*Compression stockings*: Use high-pressure compression stockings (30–40 mm Hg), worn continuously, especially during prolonged sitting or lying down, to promote venous blood return.

*Active electric movement devices (e.g., leg movement machines*): For bedridden patients, employ adjustable electric movement devices to simulate lower limb activity.

*DVT monitoring*: Closely observe postoperative changes in the patient’s lower limbs, checking daily for swelling, tenderness, and skin temperature changes. Perform lower limb ultrasound examinations on postoperative day 3 and again on day 7 to monitor for early signs of DVT.

*Patient education*: Provide detailed information to patients and their families about preventive measures for high-risk groups. Emphasize the warning signs of DVT (e.g., limb swelling, pain, difficulty breathing) and instruct patients to mobilize early after surgery, avoid prolonged sitting or standing, and maintain blood circulation.

#### 2.2.2. Routine nursing

Patients in the control group received standard nursing measures, which typically included the following:

*Early mobilization*: Patients were encouraged to perform in-bed exercises 2 to 3 days postoperatively, gradually increasing activity levels to minimize bed rest.

*Anticoagulation therapy*: Preventive anticoagulation was administered using LMWH or heparin according to standard clinical pathways, without individualized adjustments based on Caprini scores.

*Mechanical prevention*: Measures such as compression stockings or IPC devices were used, but without stratified interventions based on risk levels.

*DVT monitoring*: Routine daily palpation of the lower limbs was conducted to check for postoperative signs such as redness, swelling, or tenderness. Lower limb ultrasound examinations were performed within 3 to 5 days postoperatively.

### 2.3. Data collection

#### 2.3.1. Baseline data

Baseline demographic characteristics of patients were collected, including age, gender, body mass index (BMI), and medical history. Medical history covered chronic conditions (e.g., diabetes, hypertension, coronary heart disease), smoking history, and previous history of VTE. These data were obtained from patients’ admission records, medical archives, and imaging results, and were uniformly collected and recorded upon admission.

#### 2.3.2. Surgery-related information

Surgical details were documented, including the type of surgery (e.g., internal fixation, external fixation, joint replacement), duration of the surgery (in minutes), intraoperative blood loss (in milliliters), and the type of anesthesia used (e.g., general or regional anesthesia). This information was retrieved from surgical records and systematically organized after the surgery to analyze the impact of surgical factors on postoperative thrombosis and functional recovery.

#### 2.3.3. Caprini risk assessment

All patients underwent Caprini risk assessment, with scores recorded preoperatively, at 1 month postoperatively, and at 3 months postoperatively. These scores were used to analyze the dynamic changes in venous thrombosis risk and the effectiveness of stratified nursing interventions. The scores were calculated based on patients’ clinical characteristics and treatment records and documented in follow-up data sheets.

#### 2.3.4. Functional recovery and clinical outcomes

Postoperative functional recovery was assessed using functional scores, including the Harris Hip Score, AOFAS Ankle Score, Knee Society Score, and SF-36 Physical Component Summary (PCS). These indicators were measured and recorded before nursing interventions, at 1 month postoperatively, and at 3 months postoperatively to analyze within-group changes and between-group differences resulting from nursing interventions. Additionally, clinical outcome data, including anticoagulation-related complications (e.g., bleeding events), infections, pain, length of hospital stay, and readmission rates, were collected to comprehensively evaluate the safety and clinical benefits of stratified nursing. These data were obtained from patients’ follow-up records and hospitalization files.

### 2.4. Statistical analysis

Statistical analysis was performed using SPSS 26.0 software (IBM SPSS Statistics; IBM Corporation, Armonk). All tests were 2-tailed, with statistical significance set at *P* < .05. To reduce confounding bias, PSM was employed. Propensity scores for each patient were calculated using a logistic regression model, with matching variables including age, gender, BMI, medical history (e.g., diabetes), Caprini risk score, surgery duration, and type of anesthesia. A 1:1 nearest-neighbor matching method (caliper = 0.2) was used to achieve baseline balance between groups. Categorical variables were expressed as frequencies and percentages, and group comparisons were performed using the Chi-square test or Fisher exact test. Normally distributed continuous variables were presented as mean ± standard deviation, and non-normally distributed continuous variables as medians and interquartile ranges. Independent sample *t* tests or paired *t*-tests were used for normally distributed data, while Mann–Whitney *U* tests were used for non-normally distributed data. Survival analysis was conducted using the Kaplan–Meier method to estimate DVT-free survival time, and group differences were compared using the log-rank test. For time-series data reflecting dynamic changes, repeated measures analysis of variance was applied to assess the interaction effects of group and time factors, further validating the intervention effects of stratified nursing.

## 3. Results

### 3.1. Comparison of baseline characteristics before and after PSM

To ensure comparability of baseline characteristics between the observation group (receiving stratified nursing based on Caprini risk assessment) and the control group (receiving routine care), PSM was applied, as shown in Table 1. From the 69 patients in the control group, 51 were matched with the 51 patients in the observation group. Before matching, significant differences were observed between the 2 groups in several baseline variables: the control group had higher age (62.1 ± 11.5 years vs 56.3 ± 10.2 years, *P* = .02), BMI (26.8 ± 2.5 kg/m² vs 24.5 ± 2.3 kg/m², *P* = .01), and proportion of preoperative diabetes (22.8% vs 15.7%, *P* = .03), as well as longer surgery duration (105 ± 18 minutes vs 95 ± 15 minutes, *P* = .01), while the observation group had higher Caprini scores (5.2 ± 1.1 vs 4.6 ± 1.2, *P* = .01). After matching, these differences disappeared (*P* > .05), achieving baseline balance between the 2 groups.

**Table 1:** Baseline characteristics of experimental and control groups before and after matching.

Variable	Before matching experimental group (n = 51)	Before matching control group (n = 69)	*P*-value (before matching)	After matching experimental group (n = 51)	After matching control group (n = 51)	*P*-value (after matching)
Age (yr)	56.3 ± 10.2	62.1 ± 11.5	.02	56.3 ± 10.2	56.8 ± 10.0	.85
Gender (male%)	45.1	50.3	.15	45.1	46.2	.78
BMI (kg/m²)	24.5 ± 2.3	26.8 ± 2.5	.01	24.5 ± 2.3	24.7 ± 2.4	.8
Fracture type (femoral%)	60.8	53.6	.04	60.8	59.8	.92
Surgery duration (minutes)	95 ± 15	105 ± 18	.01	95 ± 15	96 ± 16	.88
Preoperative comorbidities (diabetes%)	15.7	22.8	.03	15.7	16.1	.89
Caprini score	5.2 ± 1.1	4.6 ± 1.2	.01	5.2 ± 1.1	5.1 ± 1.1	.78
Postoperative early mobilization (d)	2.8 ± 0.7	3.4 ± 0.8	.04	2.8 ± 0.7	2.9 ± 0.7	.87
Postoperative anticoagulation therapy (%)	80.4	72.6	.07	80.4	79.6	.91
Smoking history (%)	25.5	35.7	.02	25.5	26	.85
Multiple fractures (%)	12.8	18.6	.03	12.8	13.2	.87
Intraoperative blood loss (mL)	250 ± 50	300 ± 55	.01	250 ± 50	245 ± 48	.88
Anesthesia method (general%)	65.4	72.2	.04	65.4	65.8	.95

### 3.2. Impact of stratified nursing based on Caprini risk assessment on postoperative DVT incidence and onset time

**Figure 1.**
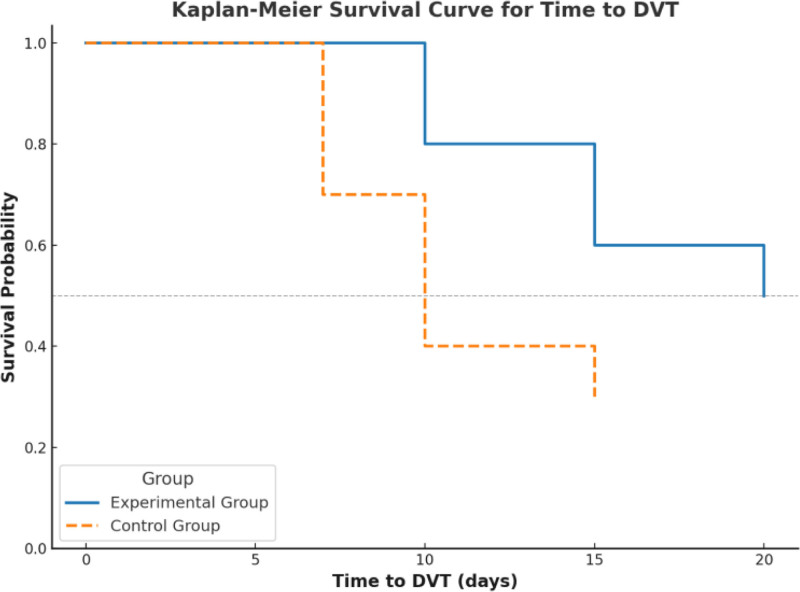
Kaplan–Meier survival curve: time to postoperative DVT in experimental and control groups. DVT = deep vein thrombosis.

There were significant differences between the observation group and the control group in terms of postoperative DVT incidence and onset time, as shown in Table 2. The DVT incidence in the observation group was 5.9% (3/51), significantly lower than 17.6% (9/51) in the control group (*P* = .04). Kaplan–Meier survival analysis revealed that the median time to DVT onset was 15 days (IQR: 12–20 days) in the observation group, which was significantly longer than 10 days (IQR: 7–15 days) in the control group (Log-Rank test, *P* = .03), indicating a longer DVT-free survival period in the observation group (Fig. 1). Additionally, the incidence of PE was 2.0% (1/51) in the observation group compared to 7.8% (4/51) in the control group, though the difference was not statistically significant (*P* = .12). These findings suggest that stratified nursing based on the Caprini risk assessment significantly reduces the incidence of postoperative DVT and delays its onset, playing a critical role in DVT prevention.

**Table 2:** Comparison of postoperative DVT incidence and time to occurrence.

Outcome	Experimental group (n = 51)	Control group (n = 51)	*P*-value
DVT incidence (%)	5.9 (3/51)	17.6 (9/51)	.04
Pulmonary embolism Incidence (%)	2.0 (1/51)	7.8 (4/51)	.12
Median time to DVT (d)	15 (IQR: 12–20)	10 (IQR: 7–15)	.03

DVT = deep vein thrombosis.

### 3.3. Comparison of DVT incidence across different Caprini risk groups

Within different Caprini risk groups, significant differences in DVT incidence were observed between the observation and control groups. In the low-risk group (Caprini score 0–2), no DVT cases occurred in the observation group (0.0%, 0/10), while the DVT incidence in the control group was 5.0% (1/20), though the difference was not statistically significant (*P* = .12), as shown in Table 3. In the moderate-risk group (Caprini score 3–4), the DVT incidence in the observation group was 5.6% (1/18), lower than 15.0% (3/20) in the control group, but the difference did not reach statistical significance (*P* = .08). In the high-risk group (Caprini score ≥ 5), the DVT incidence in the observation group was significantly lower at 10.0% (2/20) compared to 30.0% (6/20) in the control group, with the difference being statistically significant (*P* = .02). These results indicate that stratified nursing based on Caprini risk assessment is particularly effective in high-risk patients, significantly reducing the incidence of postoperative DVT. It also demonstrates a certain preventive effect in low- and moderate-risk patients, though the differences did not reach statistical significance.

**Table 3:** Subgroup analysis of DVT incidence by Caprini risk groups.

Caprini risk group	Experimental group DVT incidence (%)	Control group DVT incidence (%)	*P*-value
Low risk (0–2 points)	0.0 (0/10)	5.0 (1/20)	.12
Moderate risk (3–4 points)	5.6 (1/18)	15.0 (3/20)	.08
High risk (≥5 points)	10.0 (2/20)	30.0 (6/20)	.02

DVT = deep vein thrombosis.

### 3.4. Intra- and inter-group comparison of functional recovery

**Figure 2.**
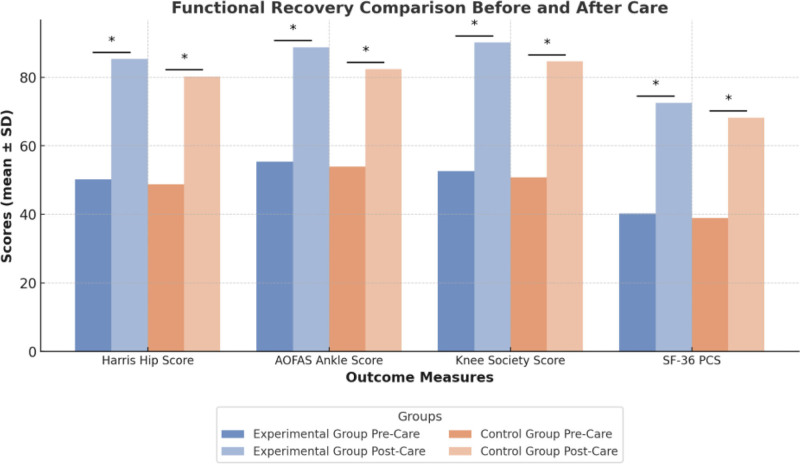
Functional recovery scores before and after care within groups.

Before nursing interventions, there were no significant differences in functional scores (Harris Hip Score, AOFAS Ankle Score, Knee Society Score, and SF-36 PCS) between the observation and control groups (*P* > .05), indicating comparable baseline functional levels between the 2 groups, as shown in Table 4. Post-nursing, both groups showed significant improvements in all functional scores. In the observation group, the Harris Hip Score increased from 50.2 ± 7.1 to 85.3 ± 5.2, the AOFAS Ankle Score improved from 55.3 ± 6.8 to 88.7 ± 4.5, the Knee Society Score rose from 52.6 ± 8.0 to 90.1 ± 6.0, and the SF-36 PCS increased from 40.2 ± 6.5 to 72.5 ± 8.3 (all *P* < .05), as illustrated in Figure 2.

**Table 4:** Group comparisons of post-care functional recovery scores.

Outcome measure	Experimental group pre-care (n = 51)	Experimental group post-care (n = 51)	Control group pre-care (n = 51)	Control group post-care (n = 51)	*P*-value
Harris Hip Score (mean ± SD)	50.2 ± 7.1	85.3 ± 5.2	48.7 ± 7.5	80.2 ± 6.5	.01
AOFAS Ankle Score (mean ± SD)	55.3 ± 6.8	88.7 ± 4.5	53.9 ± 7.2	82.4 ± 5.1	.02
Knee Society Score (mean ± SD)	52.6 ± 8.0	90.1 ± 6.0	50.8 ± 7.6	84.6 ± 7.2	.03
SF-36 Physical Component Score (PCS, mean ± SD)	40.2 ± 6.5	72.5 ± 8.3	38.9 ± 7.0	68.2 ± 9.0	.04

The control group also demonstrated significant improvements in all scores post-nursing compared to pre-nursing (*P* < .05). However, the post-nursing inter-group comparison revealed that the observation group performed significantly better than the control group in all functional scores: Harris Hip Score (85.3 ± 5.2 vs 80.2 ± 6.5, *P* = .01), AOFAS Ankle Score (88.7 ± 4.5 vs 82.4 ± 5.1, *P* = .02), Knee Society Score (90.1 ± 6.0 vs 84.6 ± 7.2, *P* = .03), and SF-36 PCS (72.5 ± 8.3 vs 68.2 ± 9.0, *P* = .04). These findings indicate that stratified nursing based on Caprini risk assessment significantly enhances postoperative functional recovery and outperforms routine nursing in all functional indicators, highlighting its clinical advantage.

### 3.5. Comparison of complications and other clinical outcomes

Significant differences were observed between the observation and control groups in terms of complications and other clinical outcomes. Among anticoagulation-related complications, the incidence of bleeding events was 2.0% (1/51) in the observation group, lower than 5.9% (3/51) in the control group, though the difference was not statistically significant (*P* = .12), as shown in Table 5. The incidence of infections was 1.9% (1/51) in the observation group and 5.8% (3/51) in the control group, with no significant difference (*P* = .15). However, the incidence of pain was significantly lower in the observation group compared to the control group (5.9% vs 13.7%, *P* = .03). The average length of hospital stay was also significantly shorter in the observation group (8.5 ± 1.2 days) compared to the control group (10.2 ± 1.5 days, *P* = .02). Additionally, the readmission rate was significantly lower in the observation group at 3.9% (2/51) compared to 9.8% (5/51) in the control group (*P* = .04). These results suggest that stratified nursing based on the Caprini risk assessment is associated with a reduction in pain, shorter hospital stays, and lower readmission rates, highlighting its clinical benefits.

**Table 5:** Comparison of complications and clinical outcomes between groups.

Outcome	Experimental group (n = 51)	Control group (n = 51)	*P*-value
Bleeding events (%)	2.0 (1/51)	5.9 (3/51)	.12
Infection rate (%)	1.9 (1/51)	5.8 (3/51)	.15
Pain incidence (%)	5.9 (3/51)	13.7 (7/51)	.03
Length of hospital stay (d, mean ± SD)	8.5 ± 1.2	10.2 ± 1.5	.02
Readmission rate (%)	3.9 (2/51)	9.8 (5/51)	.04

### 3.6. Dynamic changes in Caprini risk scores

**Figure 3.**
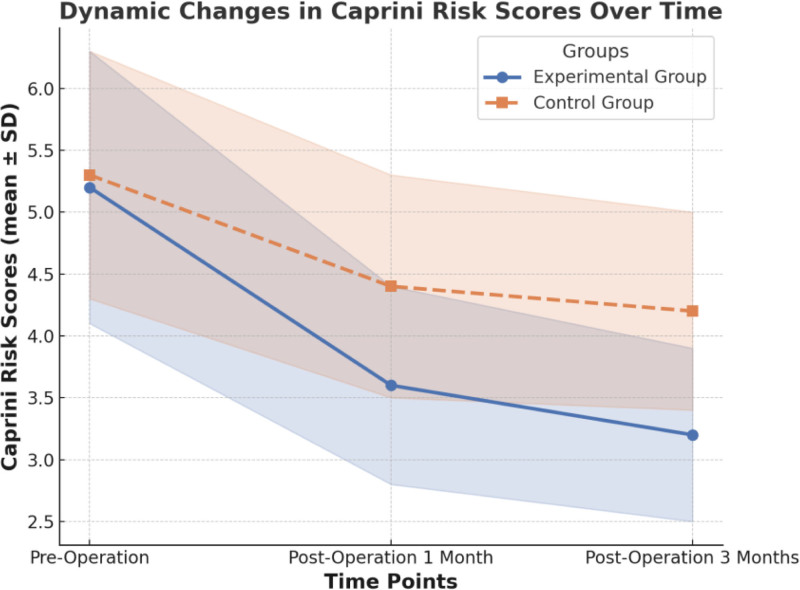
Temporal trends of Caprini risk scores with standard deviation.

There was no significant difference in preoperative Caprini risk scores between the observation group (5.2 ± 1.1) and the control group (5.3 ± 1.0, *P* = .75), indicating comparable baseline risk levels, as shown in Table 6. At 1 month postoperatively, the risk scores in the observation group significantly decreased to 3.6 ± 0.8, while the control group showed only a slight reduction to 4.4 ± 0.9, with a statistically significant difference between the groups (*P* = .02). By 3 months postoperatively, the scores in the observation group further declined to 3.2 ± 0.7, whereas the control group scores remained higher at 4.2 ± 0.8. This difference widened and remained statistically significant (*P* = .01), as illustrated in Figure 3. These findings suggest that stratified nursing based on Caprini risk assessment significantly reduces postoperative risk scores, with the effect becoming more pronounced over time. This indicates its long-term advantage in preventing postoperative thrombosis risk.

**Table 6:** Dynamic changes in Caprini risk scores over time.

Time point	Experimental group (mean ± SD)	Control group (mean ± SD)	*P*-value
Pre-operation	5.2 ± 1.1	5.3 ± 1.0	.75
Post-operation 1 mo	3.6 ± 0.8	4.4 ± 0.9	.02
Post-operation 3 mo	3.2 ± 0.7	4.2 ± 0.8	.01

## 4. Discussion

Postoperative DVT is a common and severe complication in patients with lower limb fractures. In severe cases, it can lead to life-threatening events such as pulmonary embolism, significantly impacting the recovery process. Despite the availability of various methods for DVT prevention, accurately tailoring interventions to individual risk levels remains a clinical challenge.^[12,13]^

The Caprini risk assessment tool, widely used in surgical settings, provides an effective way to evaluate the risk of postoperative DVT. Based on this, the present study implemented stratified nursing guided by Caprini scores, using PSM to minimize confounding factors, with the aim of evaluating the clinical efficacy of this model in postoperative DVT prevention.

Through retrospective analysis, this study assessed the effectiveness of stratified nursing based on the Caprini risk assessment in preventing DVT in patients with lower limb fractures. By employing PSM, the impact of potential confounding bias was reduced. The results demonstrated that stratified nursing significantly reduced the incidence of postoperative DVT, delayed its onset, and exhibited notable clinical advantages in improving functional recovery, reducing complications, and shortening hospital stays.

### 4.1. Effectiveness of Caprini risk assessment in postoperative DVT prevention

By comparing the observation group (receiving stratified nursing based on Caprini risk assessment) and the control group (receiving routine care), we found that the DVT incidence in the observation group was significantly lower than in the control group (5.9% vs 17.6%, *P* = .04). Additionally, the time to DVT onset was significantly longer in the observation group (median time: 15 days vs 10 days, *P* = .03).

These findings indicate that stratified nursing based on Caprini risk assessment effectively reduces the incidence of postoperative DVT in patients with lower limb fractures and delays its onset through individualized nursing interventions. This aligns with previous studies, confirming that the Caprini risk assessment, as a scientific and systematic tool, accurately predicts the risk of postoperative DVT and facilitates the development of personalized preventive strategies.^[14–16]^

### 4.2. Effectiveness of stratified nursing in different risk groups

In the subgroup analysis of this study, stratified nursing demonstrated varying effectiveness across different Caprini risk groups. In the low-risk group, although no DVT cases occurred in the observation group and the DVT incidence in the control group was 5.0%, the difference was not statistically significant (*P* = .12). This may be attributed to the inherently low probability of postoperative DVT in low-risk patients, resulting in minimal differences between the groups.

In the moderate-risk and high-risk groups, the DVT incidence in the observation group was significantly lower than in the control group (5.6% vs 15.0%, *P* = .08; and 10.0% vs 30.0%, *P* = .02, respectively). Notably, the effectiveness of stratified nursing was particularly prominent in the high-risk group. This can be attributed to more rigorous monitoring and targeted interventions for high-risk patients, underscoring that nursing stratification based on Caprini scores can effectively reduce the incidence of DVT, particularly in high-risk populations.^[17,18]^

### 4.3. Improvement in functional recovery

Functional recovery is a crucial indicator of postoperative rehabilitation. In terms of functional scores, patients in the observation group demonstrated significant improvements across multiple indicators, including the Harris Hip Score, AOFAS Ankle Score, Knee Society Score, and SF-36 PCS. Notably, at both 1 month and 3 months postoperatively, the functional scores in the observation group were significantly higher than those in the control group (e.g., Harris Hip Score: 85.3 ± 5.2 vs 80.2 ± 6.5, *P* = .01).

These findings suggest that stratified nursing based on Caprini risk assessment not only achieves positive outcomes in DVT prevention but also plays an important role in enhancing postoperative functional recovery. A possible mechanism is that stratified nursing interventions reduce the incidence of DVT, preventing complications caused by thrombosis and thereby providing a better environment for patient rehabilitation.^[19–21]^

### 4.4. Complications and clinical outcomes

This study also evaluated the impact of stratified nursing on anticoagulation-related complications (e.g., bleeding, infection, pain) and clinical outcomes such as length of hospital stay and readmission rates. While differences in bleeding and infection complications between the 2 groups did not reach statistical significance (*P* > .05), the incidence of pain was significantly lower in the observation group compared to the control group (5.9% vs 13.7%, *P* = .03). This suggests that stratified nursing may have certain advantages in alleviating postoperative pain.

Additionally, the average length of hospital stay was significantly shorter in the observation group (8.5 ± 1.2 days vs 10.2 ± 1.5 days, *P* = .02), and the readmission rate was also markedly lower (3.9% vs 9.8%, *P* = .04). These findings further support the advantages of stratified nursing in improving postoperative clinical outcomes, which may be directly related to its comprehensive and individualized nursing measures.^[22]^

### 4.5. Dynamic changes in Caprini risk scores

Another key finding of this study was that there was no significant difference in preoperative Caprini scores between the observation and control groups, indicating comparable baseline risks. At 1 and 3 months postoperatively, the Caprini scores in the observation group showed a marked decline, reflecting that stratified nursing effectively reduced the risk of DVT and enhanced thrombosis prevention outcomes. This dynamic change further demonstrates that the Caprini score is not only a valuable preoperative risk assessment tool but also an important reference for evaluating the effectiveness of postoperative interventions.^[23]^

### 4.6. Limitations and future directions

Although this study employed PSM to minimize bias, as a retrospective study, it is still subject to uncontrolled confounding factors, such as patient compliance and postoperative activity levels, which may have influenced the results. Additionally, the study was conducted in a single hospital with a relatively small sample size. Future research should expand the sample size and conduct prospective randomized controlled trials to validate the effectiveness of stratified nursing based on Caprini risk assessment in different hospitals and populations.

## 5. Conclusion

In summary, stratified nursing based on Caprini risk assessment is highly effective in preventing postoperative DVT in patients with lower limb fractures. It significantly reduces DVT incidence, delays its onset, improves postoperative functional recovery, and minimizes complications and hospital stays. This individualized stratified nursing model provides more precise and comprehensive postoperative management for patients with lower limb fractures, contributing to improved overall recovery outcomes.

## Author contributions

**Conceptualization:** Shuwei Huang, Yimiao Wang, Lilin Cao, Chunyan Liu.

**Data curation:** Shuwei Huang, Yimiao Wang, Lilin Cao, Chunyan Liu.

**Formal analysis:** Shuwei Huang, Yimiao Wang, Naikun Gao, Chunyan Liu.

**Funding acquisition:** Shuwei Huang, Yimiao Wang, Ruyue Wang, Chunyan Liu.

**Investigation:** Shuwei Huang, Liqun Jia, Chunyan Liu.

**Supervision:** Shuwei Huang, Yimiao Wang, Lilin Cao, Chunyan Liu.

**Visualization:** Shuwei Huang, Yimiao Wang, Chunyan Liu.

**Writing – original draft:** Shuwei Huang, Chunyan Liu.

**Writing – review & editing:** Shuwei Huang, Chunyan Liu.
